# The Characteristics of Student SARS-CoV-2 Cases on an Urban University Campus: Observational Study

**DOI:** 10.2196/39230

**Published:** 2022-09-13

**Authors:** Megan Landry, Amita Vyas, Nitasha Nagaraj, Gary A Sardon Jr, Sydney Bornstein, Hannah Latif, Padmini Kucherlapaty, Karen McDonnell, Amanda Castel, Lynn Goldman

**Affiliations:** 1 Office of the Dean Milken Institute School of Public Health The George Washington University Washington, DC United States; 2 Department of Prevention and Community Health Milken Institute School of Public Health The George Washington University Washington, DC United States; 3 Department of Epidemiology Milken Institute School of Public Health The George Washington University Washington, DC United States; 4 Department of Environmental and Occupational Health Milken Institute School of Public Health The George Washington University Washington, DC United States

**Keywords:** COVID-19, SARS-CoV-2, college, university, students, young adult, youth, communicable disease, prevention, school health, outbreak prevention, contact tracing, pandemic

## Abstract

**Background:**

Academic institutions are central hubs for young adults, laden with academic and social interactions and communal living arrangements, heightening the risk of transmission of many communicable diseases, including COVID-19. Shortly after the start of the fall 2020 academic year, institutions of higher learning were identified as hot spots for rises in COVID-19 incidence among young adults.

**Objective:**

This study aims to identify the characteristics of student SARS-CoV-2 cases, identify the extent to which the student population adhered to preventative strategies, and examine behaviors that would put them at higher risk of contracting or spreading COVID-19.

**Methods:**

This observational study comprises 1175 university students at The George Washington University in Washington, DC, with a confirmed COVID-19 diagnosis between August 3, 2020, and November 30, 2021. Case investigation and contact tracing tools were developed by the Campus COVID-19 Support Team and captured in REDCap (Research Electronic Data Capture). Trained case investigators were notified of a case and attempted to contact all cases within 24 hours of the case receiving their lab result. Associations between case characteristics and number of contacts were examined using Wilcoxon rank sum tests. Knowledge of exposure, behaviors since exposure, student residence status, and fraternity and sorority life affiliation were examined using chi-square tests.

**Results:**

Positive student cases reported a median of 3 close contacts, and 84.6% (993/1175) reported at least one symptom with a median of 4 COVID-19 symptoms. Congestion (628/1175, 53.4%), cough (530/1175, 45.1%), and headache (484/1175, 41.2%) were the most frequently reported symptoms. Moreover, 36% (415/1160) reported that they did not know how they were exposed to the virus. Among those aware of contact with a COVID-19 confirmed case, 55.1% (109/198) reported the contact was a close friend or family member, and 25.3% (50/198) reported that it was someone with whom they lived. Athlete (vs nonathlete; *P*<.001), on-campus (vs off-campus; *P*<.001), and undergraduate (vs graduate; *P*=.01) students all reported a significantly higher number of contacts. Students living on campus were more likely to report attending campus events in the 2 days prior to symptom onset or positive test result (*P*=.004). Students with fraternity or sorority affiliation were more likely to report attending campus events in the 2 days prior to symptom onset or positive test result (*P*<.001).

**Conclusions:**

COVID-19 cases have not yet stabilized to a predictable state, but this study provides case characteristics and insights for how academic institutions might prepare to mitigate outbreaks on their campuses as the world plans for the transition from pandemic to endemic COVID-19.

## Introduction

The world has witnessed major disruption in all domains of life since the World Health Organization declared the novel SARS-CoV-2 outbreak a global pandemic in March 2020. Although children and young adults were initially reported to be at lower risk for severe disease or death from COVID-19, emerging evidence suggested a rise in cases among 18- to 22-year-olds [1]. Academic institutions are central hubs for young adults of this age group, and the congregate settings, academic and social interactions, and communal living arrangements inherent to college heighten the risk of transmission of many communicable diseases, including COVID-19 [2].

In spring 2020, colleges and universities across the United States transitioned to virtual learning to curb the transmission of SARS-CoV-2. However, for the fall 2020 semester, several institutions returned to some form of in-person instruction or campus living. By September 2020, 4% of colleges were conducting full in-person instruction, 23% were primarily in person, while the remaining majority implemented remote or hybrid learning [3]. Shortly after the start of the fall 2020 academic year, institutions of higher learning were identified as hot spots for rises in COVID-19 incidence among young adults [4]. A county-level analysis comparing 21-day periods before and after classes began in fall 2020 revealed that large colleges and universities with remote instruction observed a 17.9% decrease in COVID-19 incidence, and those with in-person instruction experienced a 56% increase in COVID-19 incidence [5].

Evidence is suggestive of young adults being less likely to adhere to COVID-19 preventive measures than any other age group [1,6]. Additionally, a traditional on-campus academic experience typically includes extracurricular group activities and social gatherings, which are difficult to monitor for adherence to public health measures to prevent outbreaks. During August to September 2020, a university in Arkansas reported 965 cases, with 31% of these cases reporting involvement in fraternity and sorority activity; 91% of the gatherings identified in network analysis were linked by participation in fraternity and sorority activities [7]. SARS-CoV-2 can also spread quickly among college athletes [8], and outbreaks among university sports teams have received a great deal of media attention; however, the extent to which these are due to contact during sports activities is unclear. For an outbreak among men’s and women’s soccer teams in a university, 73% of the cases were living in shared housing, and approximately 60% attended at least one social gathering, including a birthday party, visits to friends’ dormitories or apartments, and outdoor lake gatherings [9].

The George Washington University (GWU), an urban campus with its largest campus in Washington, DC, prepared for a limited reopening of its campus in fall 2020 and put into place multiple strategies to mitigate the spread of SARS-CoV-2 including the following: (1) requiring physical distancing and face coverings and limiting gatherings to less than 10 people; (2) return to campus monitoring and testing for on-campus residential students on arrival and 5 days later, including quarantine of residence hall students pending 2 negative polymerase chain reaction (PCR) tests; (3) mandatory weekly SARS-CoV-2 PCR testing for on-campus students, faculty, and staff, and thrice weekly testing for athletes; (4) mandatory daily symptom monitoring for all on-campus students, faculty, and staff; (5) on-campus investigations to quickly identify transmission pathways and intervene early via quarantine and testing of suspected close contacts of the cases; (6) clinical follow-up, quarantine, and testing at any point for any member of the campus community who develops symptoms; (7) per rules of the District of Columbia Health Department, a 14-day quarantine of anyone returning to campus from states defined by them as “high risk”; and (8) mandatory influenza vaccine [10,11].

This analysis aims to identify the characteristics of student SARS-CoV-2 cases at GWU, identify the extent to which the student population adhered to preventative strategies, and examine health behaviors that would put them at higher risk of contracting or spreading COVID-19. Lessons learned can contribute to refining COVID-19 protocols and pandemic programming in academic institutions.

## Methods

### Participants and Data Collection

This observational study comprises 1175 university students at GWU with a confirmed COVID-19 diagnosis between August 3, 2020, and November 30, 2021. GWU has over 26,000 undergraduate and graduate students, yet most of these individuals were learning remotely during the 2020-2021 academic year. Individuals authorized to be on campus during the fall 2020 and spring 2021 semesters were required to participate in weekly surveillance testing and to complete daily symptom screening surveys [11]. The student population approved to live in residence halls on campus or having access to campus was identified based on enrollment (in a small number of classes that continued to be delivered in person) and active ongoing research; student athletes; hardship students with limited alternative housing resources; or being employed as student workers in essential on-campus jobs. The on-campus student population that was required to participate in the weekly surveillance program comprised approximately 500 individuals in fall 2020 and increased to approximately 2500 individuals in spring 2021 owing to reopening more residence hall space, as well as increased on-campus instruction and research. In addition, starting in October 2020, approximately 13,000 students who were not authorized to be on campus (ie, students who lived near GWU) were able to access voluntary testing for travel or if they were experiencing symptoms. By fall 2021, the university had welcomed back more than 20,000 in-person students who were required to participate in twice-monthly surveillance testing. Surveillance testing, contract tracing, and outbreak response strategies are described elsewhere [10,11].

Case investigation and contact tracing tools were developed by the GWU Campus COVID-19 Support Team, comprised of public health experts. All data were captured in REDCap (Research Electronic Data Capture), a secure web application for web-based surveys and databases [12]. Trained GWU case investigators, or contact tracers, were notified of a case and attempted to contact all cases within 24 hours of the case receiving their result. A single case investigation took approximately 30 minutes to complete, soliciting information on case demographics, symptoms, underlying health conditions, risk-reduction behaviors, known exposures within the 48 hours before symptom onset or a positive test, campus authorization, and campus affiliation.

### Statistical Analysis

We used univariate analyses to examine the frequencies and percentages of categorical variables, as well as medians and distributions of continuous variables. We examined the bivariate relationships between selected case characteristics and number of contacts using Wilcoxon rank sum tests. Additionally, bivariate relationships were examined between knowledge of exposure and behaviors since exposure, student residence status and knowledge and behaviors since exposure, and fraternity and sorority life affiliation using chi-square tests. All hypothesis tests were 2-sided, and the level of significance was set to an alpha of .05. Analyses were performed using SAS, version 9.4 (SAS Institute Inc).

### Ethical Considerations

All students provided informed consent to participate in the GWU COVID-19 surveillance program, and the GWU Institutional Review Board concluded that these were non–research-related activities.

## Results

Between August 3, 2020, and November 30, 2021, the university public health lab performed 219,919 SARS-CoV-2 PCR tests for our student population, resulting in 1175 cases, which represents 84% (1175/1399) of the total university cases during the study period. Table 1 presents the characteristics of students found to be infected with SARS-CoV-2. The median age of students who tested positive for COVID-19 was 21 years. Moreover, out of the 1175 student cases, 831 (70.7%) cases were White, 707 (60.2%) self-identified as female, 877 (74.7%) were undergraduates, 862 (73.4%) lived off campus, and 945 (80.4%) reported as never smoking. Only 92 (8%) cases were student athletes, and 337 (28.7%) were affiliated with fraternity or sorority life. Students who tested positive for COVID-19 reported a median of 3 close contacts. Nearly 85% (993/1175) of students reported at least one symptom with a median of 4 COVID-19 associated symptoms being reported.

**Figure 1:**
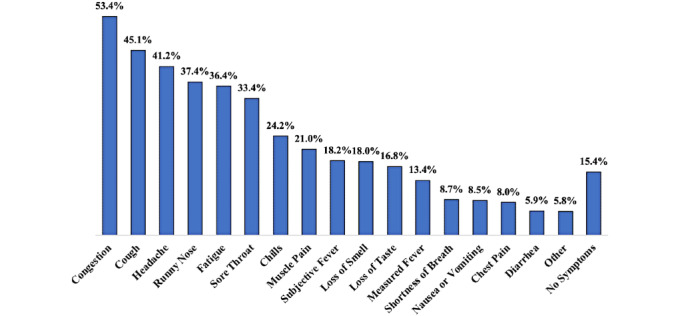
Frequency of reported symptoms among student COVID-19 cases (N=1175).

The top 3 most reported symptoms among the 1175 cases were 628 (53.4%) reporting congestion, 530 (45.1%) reporting cough, and 484 (41.2%) reporting headache. Just over 15% (n=181) of cases reported experiencing no symptoms (Figure 1). Underlying health conditions were somewhat common, with 224 (19.1%) students reporting one or more underlying medical conditions that put them at higher risk for COVID-19 complications.

Table 2 presents knowledge and behaviors 48 hours prior to testing positive. Out of the 1160 cases, 415 (35.8%) reported they did not know how they were exposed to the virus. Of the 198 cases aware of prior contact with a COVID-19 confirmed case, 109 (55.1%) reported the contact was a close friend or family member, and 50 (25%) reported that it was someone with whom they lived. When asked about preventative health behaviors 48 hours prior to the onset of symptoms or positive test result, 44 (5%) out of 846 reported not having used personal protective equipment (PPE) such as masks on campus. In addition, of 1159 cases, 242 (20.9%) reported domestic travel in the 2 days prior to symptom onset or their positive test result. Moreover, 402 (35.6%) of 1128 cases reported using local transport in the form of city buses, city trains, or ride sharing services such as Lyft or Uber. Additionally, 251 (21.4%) out of 1175 reported being around people from campus in the 2 days prior to symptom onset or their positive test result.

Table 3 presents the median number of contacts by select case characteristics. Athletes reported a median of 5 (IQR 3-11) contacts compared to 3 (IQR 1-5) for nonathletes (*P*<.001). Students living on campus reported a median of 4 (IQR 2-7) contacts compared to 3 (IQR 1-4) reported by those living off campus (*P*<.001). Undergraduate students reported a median of 3 (IQR 2-6) contacts compared to 3 (IQR 1-4) reported by graduate students (*P*=.01). There were no significant differences in the number of contacts by self-reported gender identity or fraternity and sorority life (FSL) affiliation.

**Table 1:** Case characteristics of students (N=1175).

Characteristics		Values
Age (years), median (IQR)		21 (20-23)
**Gender, n (%)**		
	Woman	707 (60.2)
	Man	456 (38.8)
	Non–gender conforming	8 (0.7)
	Missing	4 (0.3)
**Race, n (%)**		
	Asian	123 (10.5)
	Black or African American	97 (8.3)
	Multiracial	44 (3.7)
	Native Hawaiian or other Pacific Islander	1 (0.1)
	White	831 (70.7)
	Other	76 (6.5)
	Missing	3 (0.3)
**Student status, n (%)**		
	Undergraduate	877 (74.7)
	Graduate	278 (23.7)
	Other	16 (1.4)
	Missing	4 (0.3)
**Athlete, n (%)**		
	Yes	92 (7.8)
	No	1083 (92.2)
**Fraternity and sorority life, n (%)**		
	Yes	337 (28.7)
	No	838 (71.3)
**Student residence, n (%)**		
	On campus	313 (26.6)
	Off campus	862 (73.4)
**Smoking status, n (%)**		
	Current	122 (10.4)
	Former	89 (7.6)
	Never	945 (80.4)
	Missing	19 (1.6)
Number of contacts, median (IQR)^a^		3 (2-5)
Number of symptoms, median (IQR)		4 (2-6)
**Underlying conditions, n (%)**		
	None	907 (77.2)
	One or more	224 (19.1)
	Missing	44 (3.7)

^a^N=837.

**Table 2:** Knowledge and behaviors prior to a positive COVID-19 test.

Knowledge and behaviors		Values, n (%)
Knowledge of exposure (n=1160)		745 (64.2)
**Prior contact with confirmed case (n=198)**		
	A close friend or family	109 (55.1)
	Classmate	8 (4.0)
	Coworker	5 (2.5)
	Someone you live with	50 (25.3)
	Other^a^	23 (11.6)
	Missing	3 (1.5)
Traveled within the United States 2 days prior to symptom onset or test date (n=1159)		242 (20.9)
Used any local transportation 2 days prior to symptom onset or test date (n=1128)		402 (35.6)
Did not use any PPE^b^ on campus 2 days prior to symptom onset or positive test result (n=846)		44 (5.2)
Were around other people on campus 2 days prior to symptoms starting or positive test results (ie, training, conferences, concerts, parties, dinner, etc; n=1175)		251 (21.4)

^a^Other includes those met during travel, friend of friends, and teammate.

^b^PPE: personal protective equipment (eg, masks, hand-made masks, and gloves).

**Table 3:** Median number of contacts by select case characteristics.

Characteristics		Median (IQR)	*P* value
**Gender (n=826)**			.61
	Woman	3 (2-5)	
	Man	3 (2-5)	
**Student status (n=826)**			.01
	Undergraduate	3 (2-6)	
	Graduate	3 (1-4)	
**Athlete (n=837)**			<.001
	No	3 (1-5)	
	Yes	5 (3-11)	
**Fraternity or sorority (n=837)**			.55
	No	3 (1-5)	
	Yes	3 (2-5)	
**Student residence (n=837)**			<.001
	On campus	4 (2-7)	
	Off campus	3 (1-4)	

Table 4 presents exposures and behaviors by student residence (on campus versus off campus). Students living on campus were more likely to report using any PPE (eg, masks, hand-made masks, and gloves) on campus in the 2 days prior to symptom onset or positive test result compared to students living off campus (*P*=.006). Not surprisingly, students who lived on campus were more likely to report attending campus events in the 2 days prior to symptom onset or positive test result (*P*=.004). There were no statistically significant differences between living on campus or off campus in terms of domestic travel, local transportation use, or knowledge of exposure in the 2 days prior to symptom onset or positive test result.

Table 5 presents student exposures and behaviors by FSL affiliation. There were no statistically significant differences between FSL affiliation in terms of knowledge of exposure or using PPE in the 2 days prior to symptom onset or positive test result. Those reporting no FSL affiliation were more likely to report using domestic travel or local transportation in the 2 days prior to symptom onset or positive test result (*P*<.001 and *P*<.001, respectively). However, students reporting an FSL affiliation were more likely to report attending campus events (*P*<.001) in the 2 days prior to symptom onset or positive test result.

**Table 4:** Exposure and behaviors by student residence.

Exposure and behaviors			Off campus, n (%)		On campus, n (%)		*P* value	
**Knowledge of exposure (n=1160)**								.06
	Yes	562 (65.8)		183 (59.8)				
	No	292 (34.2)		123 (40.2)				
**Used any PPE^a^ (n=846)**								.006
	Yes	554 (93.4)		248 (98.0)				
	No	39 (6.6)		5 (2.0)				
**Travel within the United States (n=1159)**								.33
	Yes	183 (21.6)		59 (19.0)				
	No	665 (78.4)		252 (81.0)				
**Used local transportation (n=1128)**								.33
	Yes	286 (34.8)		116 (37.9)				
	No	536 (65.2)		190 (62.1)				
**Attended any campus events (n=1042)**								.004
	Yes	282 (37.0)		131 (47.0)				
	No	481 (63.0)		148 (53.0)				

^a^PPE: personal protective equipment.

**Table 5:** Exposure and behaviors by fraternity and sorority life (FSL) affiliation.

Exposure and behaviors		Non-FSL, n (%)	FSL, n (%)	*P* value	
**Knowledge of exposure (n=1160)**					.17
	Yes	519 (63.0)	226 (67.3)		
	No	305 (37.0)	110 (32.7)		
**Used any PPE^a^ (n=846)**					.61
	Yes	573 (94.6)	229 (95.4)		
	No	33 (5.4)	11 (4.6)		
**Travel within the United States (n=1159)**					<.001
	Yes	201 (24.3)	41 (12.2)		
	No	623 (75.6)	294 (87.8)		
**Used local transportation (n=1128)**					<.001
	Yes	323 (40.2)	79 (24.4)		
	No	481 (59.8)	245 (75.6)		
**Attended any campus events (n=1042)**					<.001
	Yes	262 (35.2)	151 (50.7)		
	No	482 (64.8)	147 (49.3)		

^a^PPE: personal protective equipment.

## Discussion

### Principal Findings

The findings from this study contribute to the limited understanding of how COVID-19 uniquely presents itself in a population of university students by describing the case characteristics, risk and protective behaviors, and adherence to preventive strategies among students on a large university campus. Most students in this analysis reported being aware of their exposure and reported that the exposure resulted from a close friend or family member outside of an academic setting. It should be noted that, to date, we have not identified any significant, unexplained classroom transmission, which is suggestive that indoor masking, along with rigorous case investigations, contact tracing, and surveillance testing were successful in preventing widespread transmission within classrooms. This is an especially important finding as campuses weigh web-based or hybrid learning during future COVID-19 surges. Additionally, athletes reported a higher number of contacts, while on-campus students, along with students affiliated with FSL, reported that they were more likely to attend events, potentially increasing their own exposure risk or exposing others.

### Comparison With Prior Work

Our results are consistent with previous research that suggests extracurricular groups and social gatherings such as fraternity and sorority activities can be hot spots for spreading SARS-CoV-2 [7]. These groups are traditionally together a lot, and by nature this could put them at higher risk. We observed FSL affiliates more likely to attend social events, and it is difficult to monitor adherence to public health measures during these events. To help reduce spread among extracurricular group activities, public health campaigns should be designed in concert with and specifically for such groups (ie, athletes and FSL leaders) where gathering or close contact is sometimes necessary during interactions. Additionally, increased testing of specific groups (eg, athletes or FSL members) during periods of high transmission should be implemented to quickly detect a rise in cases and move individuals into isolation or quarantine to mitigate further spread. Public health ambassadors can be deployed to work specifically with these groups to promote risk reduction behaviors, monitor results, and mitigate spread if outbreaks are detected.

It has been suggested that riskier behaviors and certain settings, such as unmasked events or parties and communal living with limited space to social distance, place college campuses at greater risk for outbreaks of COVID-19 and becoming “super spreaders” for neighboring communities [8]. Despite exposure locale, we recognize that college campuses can harbor “super spreader” situations due to college settings encompassing groups of individuals that potentially spend a lot of time together, such as athletes, FSL affiliates, and communal living spaces where transmission can be difficult to contain. Thus, primary prevention efforts during waves of high transmission must be a cornerstone of campus safety to mitigate the spread. This should include the following: strong support and continuous communication for greater adherence to indoor masking in campus buildings; increased ventilation; having proper hand hygiene; carrying out social distancing where possible; eliminating buffets in dining halls; de-densifying campuses and classrooms; and frequent cleaning to further mitigate the spread of the virus within classrooms and academic settings [13,14].

### Strengths and Limitations

While this study provides insights into COVID-19 case characteristics on an urban university campus, there are limitations that should be considered before generalizing to other university or college settings. First, there is potential bias from cases self-reporting underlying conditions, behaviors, and perceived symptoms. To mitigate self-reporting bias, we used prompts, listed out and read a list of underlying conditions and symptoms, and provided important dates during the interview for recalling behaviors during the interviewees’ infectious period or when symptoms may have started. Second, our survey evolved over the course of the pandemic, and some study questions, such as participation in FSL, were not asked until later versions of the survey. To address FSL affiliation for cases prior to fall 2021, we obtained a roster from our division of student affairs, which may have included some discrepancies of people not continuing in FSL for a whole semester. Third, our study participants may have also experienced recall bias or social desirability bias concerning their behaviors before becoming infected with SARS-CoV-2. To mitigate any potential social desirability bias, we reviewed statements of confidentiality with cases and reminded them throughout the interview that their answers would not impact their standing at the university in any way. Finally, our sample was predominately White and female, which corresponds to the university’s demographics of 77.2% White (n=20,849) and 60.7% female (n=16,798). However, we acknowledge that these results are not generalizable to a larger population, and results should be interpreted with caution before generalizing to other populations.

### Future Directions

Despite many colleges and universities requiring the COVID-19 vaccine prior to the fall 2021 semester, we must continue primary prevention efforts and testing, accompanied by isolation of cases and quarantine of individuals not up to date with current vaccines, to reduce the spread of variants of concern and subsequent further mutations [15]. The weekly surveillance measures put into place for this study setting were instrumental in maintaining a relatively low daily positivity rate for the university community. During the study period, the daily positivity rate at GWU ranged between 0.00% and 5.45%, whereas the larger Washington, DC daily positivity rate reached 24.6% [16]. As an urban college campus, it is our responsibility to strive to keep our students and the larger community safe and make concerted efforts to mitigate COVID-19 transmission on our campuses [17]. We can continue to provide a traditional college experience, and we can remain in classrooms together, but we must implement masking, testing, and other risk reduction procedures to keep our campuses safe.

### Conclusions

Along with its devastating health outcomes, COVID-19 also sparked an *education pandemic* with lockdowns disrupting the lives of children, adolescents, and young adults. While much attention has been placed on K-12 education and SARS-CoV-2 transmission, less is known about the college or university context. Further, as the world continues to experience repeated outbreaks of different COVID-19 variants, it underscores the importance of understanding the behaviors of students on a college campus. COVID-19 cases have not yet become stabilized to a predictable state, but this analysis provides case characteristics and insights for how academic institutions might prepare to mitigate outbreaks on their campuses as the world plans for the transition from pandemic to endemic COVID-19 and having to deal with evolving variants. It is imperative that colleges and universities continue to plan for waves of increased COVID-19 transmission on campuses and have surge capacity in place to maintain a certain level of operational procedures during times of increased transmission. Faculty must be prepared to adapt instruction to account for themselves or their students potentially missing up to 10 days of classes.

## Abbreviations

FSL: fraternity and sorority life
GWU: The George Washington University
PCR: polymerase chain reaction
PPE: personal protective equipment
REDCap: Research Electronic Data Capture

## Data Availability

The data sets generated or analyzed during this study are available from the corresponding author on reasonable request.
